# Supplementary material for: Virtual Screening of Artemisia annua Phytochemicals as Potential Inhibitors of SARS-CoV-2 Main Protease Enzyme
Source: Molecules. 2022 Nov 21;27(22):8103. doi: 10.3390/molecules27228103 (PMC9695405; doi:10.3390/molecules27228103)
Supplement: Supplementary file 1 [file molecules-27-08103-s001.zip › molecules-1989454-supplementary.pdf]

Supplementary file

Virtual Screening of *Artemisia annua* Phytochemicals as Potential Inhibitors of SARS-CoV-2 Main Protease Enzyme

Khalid Miandad [1], Asad Ullah [1], Kashif Bashir [1], Saifullah Khan [2], Syed Ainul Abideen [3], Bilal Shaker [4], Metab Alharbi [5], Abdulrahman Alshammari [5] , Mahwish Ali [6], Abdul Haleem [7] and Sajjad Ahmad [1,*]

[1] Department of Health and Biological Sciences, Abasyn University, Peshawar 25000, Pakistan
[2] Institute of Biotechnology and Microbiology, Bacha Khan University, Charsadda, Pakistan
[3] School of Biomedical Engineering, Shanghai Jiao Tong University, China
[4] Department of Biomedical Engineering, Chung-Ang University, Seoul, South Korea
[5] Department of Pharmacology and Toxicology, College of Pharmacy, King Saud University, P.O. Box 2455, Riyadh 11451, Saudi Arabia
[6] Department of Biological Science, National University of Medical Sciences, Rawalpindi 46000, Pakistan
[7] Department of Microbiology, Quaid-i-Azam University, Islamabad, Pakistan
* Correspondence: sajjad.ahmad@abasyn.edu.pk

Table S1. Physiochemical properties and pharmacokinetics analysis (ADME) of Top-3 complexes.

Compounds	Top-1	Top-2	Top-3
	**Physicochemical Properties**		

Formula	$C_{13}H_{14}N_3O_4S$	$C_{12}H_{10}N_2O_4$	$C_{13}H_{16}N_2O_4$
Molecular weight	308.33 g/mol	246.22 g/mol	264.28 g/mol
Num. heavy atoms	21	18	19
Num. arom. .heavy atoms	11	11	6
Fraction Csp3	0.23	0.08	0.46
Num. rotatable bonds	5	4	5
Num. H-bond acceptors	4	4	4
Num. H-bond donors	3	1	2
Molar Refractivity	77.46	61.13	66.85
TPSA	135.72 Å²	86.24 Å²	93.34 Å²
	Lipophilicity		
Log Po/w (iLOGP)	-4.96	-0.97	0.66
Log Po/w (XLOGP3)	1.84	0.94	1.20
Log Po/w (WLOGP)	0.76	0.96	0.87
Log Po/w (MLOGP)	0.30	0.56	0.63
Log Po/w (SILICOS-IT)	1.88	-0.21	0.39
Consensus Log Po/w	-0.04	0.26	0.75

	Water Solubility		
Log S (ESOL)	-2.97	-2.15	-2.14
Solubility	3.32e-01 mg/ml ; 1.08e-03 mol/l	1.76e+00 mg/ml ; 7.13e-03 mol/l	1.92e+00 mg/ml ; 7.27e-03 mol/l
Class	Soluble	Soluble	Soluble
Log S (Ali)	-4.31	-2.34	-2.76
Solubility	1.51e-02 mg/ml ;4.89e-05 mol/l	1.13e+00 mg/ml ; 4.60e-03 mol/l	4.63e-01 mg/ml ; 1.75e-03 mol/l
Class	Moderately soluble	Soluble	Soluble
Log S (SILICOS-IT)	-2.58	-1.26	-1.57
Solubility	8.04e-01 mg/ml ; 2.61e-03 mol/l	1.34e+01 mg/ml ; 5.46e-02 mol/l	7.16e+00 mg/ml ; 2.71e-02 mol/l
Class	Soluble	Soluble	Soluble
	Pharmacokinetics		
GI absorption	High	High	High
BBB permeant	No	No	No
P-gp substrate	Yes	No	No
CYP1A2 inhibitor	Yes	No	No
CYP2C19 inhibitor	No	No	No
CYP2C9 inhibitor	No	No	No

CYP2D6 inhibitor	No	No	No
CYP3A4 inhibitor	No	No	No
Log Kp (skin permeation)	-6.87cm/s	-7.13 cm/s	-7.06 cm/s
	Drug-likeness		
Lipinski	Yes; 0 violation	Yes; 0 violation	Yes; 0 violation
Ghose	Yes	Yes	Yes
Veber	Yes	Yes	Yes
Egan	No; 1 violation: TPSA>131.6	Yes	Yes
Muegge	Yes	Yes	Yes
Bioavailability Score	0.55	0.56	0.56
	Medicinal Chemistry		
PAINS	0 alert	0 alert	0 alert
Brenk	1 alert: quaternary_nitrogen_1	1 alert: quaternary_nitrogen_1	1 alert: quaternary_nitrogen_1
Lead-likeness	Yes	No; 1 violation: MW<250	Yes
Synthetic accessibility	2.82	2.19	2.27
